# Establishment of an *in-vitro* inflammatory bowel disease model using immunological differentiation of Caco-2 cells

**DOI:** 10.1016/j.mex.2024.102952

**Published:** 2024-09-13

**Authors:** Ippei Uemura, Natsuko Takahashi-Suzuki, Fumiya Kita, Takashi Satoh

**Affiliations:** Department of Pharmaceutics, Faculty of Pharmaceutical Sciences, Hokkaido University of Science, 7-Jo 15-4-1 Maeda, Teine-ku, Sapporo, Hokkaido 006-8585, Japan

**Keywords:** Α-defensin5, Inflammatory bowel disease, Sodium dextran sulfate, Intestinal immunity, In-vitro inflammatory bowel disease model using immunological differentiation of Caco-2 cells

## Abstract

Studies on intestinal cell differentiation, particularly in dextran sodium sulfate (DSS)-induced inflammatory bowel disease (IBD), have predominantly focused on the disruption of intestinal crypts and suppressive effects on the intestinal microbiota; however, repeated administration of DSS is required to induce inflammatory pathology, and there is a lack of observation of early responses and consideration of differentiation stages. Although colonic adenocarcinoma (Caco-2) cells can be used as intestinal cell models, research on these cells in an immature state is limited. We, therefore, investigated the relationship between Caco-2 cell culture duration and immunological differentiation using α-defensin5 (DEFA5) as an indicator of intestinal immunity and differentiation. Changes in protein and gene expression levels in response to DSS were examined at each differentiation stage. Expression of immune- and differentiation-related proteins, including DEFA5 and lysozyme, was evident from Day 8 of culture. Immune responses to DSS varied with the differentiation stage, affecting cell viability and cytokine expression.

• Caco-2 cell culture duration correlates with the differentiation stage of Paneth cells.
• DSS exposure elicits different effects depending on the differentiation stage.
• Our *in-vitro* model of IBD facilitates the characterization of the cell differentiation process and provides a methodology to help elucidate the causal mechanisms of IBD.

## Graphical abstract

**Figure:**
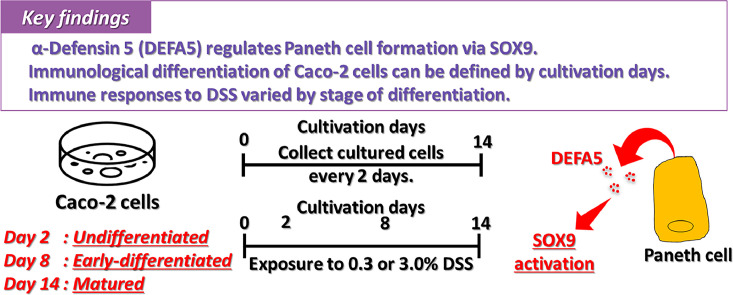

Specifications table

**Table:** 

Subject area:	Immunology and Microbiology
More specific subject area:	Inflammatory diseases, Experimental model
Name of your method:	In-vitro inflammatory bowel disease model using immunological differentiation of Caco-2 cells
Name and reference of original method:	Not applicable.
Resource availability:	Resource sources are included within the text.

## Background

The role of undifferentiated cells, such as leucine-rich repeat-containing G protein-coupled receptor 5 (LGR5)-positive intestinal stem cells, in inflammatory bowel disease (IBD) development, is recognized, particularly within intestinal crypts where DEFA5-expressing Paneth cells are concentrated. However, data regarding the effect of receptor mutations on antimicrobial peptide expression and differentiation and maturation processes are limited [[1], [2], [3]]. Despite the use of animal models in inflammation-related research, assessing the effect of inflammatory agents on various cell differentiation stages throughout the intestine poses is complex [4]. In IBD models induced using dextran sulfate sodium (DSS), the standard acute colitis model typically uses 2%–3% DSS for 5–7 days and the chronic model recommends multiple cycles of DSS exposure [[3], [4], [5]], but it remains challenging in both cases to evaluate the initial defense responses centered on innate immunity induced by a single administration and to distinguish the differences in reactivity between mature and immature differentiation. While animal models are useful for research aimed at suppressing inflammation, it is essential to consider the cell differentiation process to inform the development of new therapeutic targets for IBD pathogenesis.

Colonic adenocarcinoma (Caco-2) cells are commonly used as a pharmacokinetic predictive model. In addition, they can be used to assess host defense mechanisms in intestinal innate immunity, particularly using DEFA5 derived from intestinal Paneth cells [[6], [7], [8], [9], [10]]. The flexibility in varying passage numbers and culture duration makes Caco-2 cells highly adaptable for *in-vitro* research [11,12]. Indeed, changes in intestinal cell differentiation have been associated with variations in culture duration [6,11]. Notably, Caco-2 cells differentiate into small intestinal epithelial-like cells after 14 days of culture [7,9,12]; however, most studies using these cells have focused on differentiated cells; thus, respective results on their immature state are scarce. Meanwhile, recent studies have investigated the effects of oral anticancer drugs and food components on the gut immune system using Caco-2 cells and reported their complete differentiation by the end of 14-day cultures [[6], [7], [8], [9], [10]]. Moreover, the anti-inflammatory properties of gut-derived secretions support the relevance of using differentiation factors to further elucidate the intricacies of the intestinal immune system [[13], [14], [15], [16]].

Caco-2 cells can serve as a valuable tool for elucidating the dynamics of Paneth cell differentiation and its implications for intestinal immunity [6]. The interaction between Wnt signaling and Yes1-associated transcriptional regulator pathways is crucial for LGR5-positive intestinal stem cells to differentiate into Paneth cells [[17], [18], [19]]. The presence of SRY-box transcription factor 9 is also essential to this process [17]. We hypothesized that these differentiation markers, with Paneth cell-derived DEFA5 and lysozyme, can be used to track the immunological differentiation of Caco-2 cells.

DSS-induced IBD involves gut bacteria producing hydrogen sulfide, leading to cell damage, inflammation, and cell cycle arrest; however, the effect of the cell differentiation stage remains unclear in studies using fully differentiated Caco-2 cells [[11], [12], [13]]. Meanwhile, studies on DSS-exposed cells have been limited to their use as a supplement to *in vivo* experiments through knockdown experiments and to assess cytokine effects [[20], [21], [22], [23], [24], [25], [26]]. We thus conducted this study to monitor immune- and differentiation-related indicators in Caco-2 cells to assess responses to DSS exposure across cell differentiation stages and to establish an *in-vitro* model of DSS-induced IBD considering the differentiation process.

## Method details

### Chemicals

DSS (36–50 kDa) was purchased from MP Biomedicals (Tokyo, Japan). DEFA5 was purchased from Peptide Institute, Inc. (Osaka, Japan).

### Cell culture and treatment

Caco-2 cells were obtained from the European Collection of Authenticated Cell Cultures (Salisbury, UK; passages 19–36). The culture conditions were established as previously reported [10]. When cells reached 100 % confluency, trypsin was added, incubated at 37 °C for 5 min, and centrifuged at 1000 rpm for 5 min. The trypsin was then removed and the cells were resuspended using a dropper to create a suspension solution with a cell density of 1.0 × 10^6^ cells/mL. The cells were seeded into new flasks at 1.0 × 10^4^ cells/mL for one cycle of passaging and subsequently seeded into culture plates at 1.0 × 10^4^ cells/mL (2.0 mL/well for 6-well plates, 1.0 mL/well for 12-well plates, and 0.5 mL/well for 24-well plates). The use of plates with as large an area as possible is recommended, particularly during days 2 and 4 of culture, when cell numbers are low, and cell recovery may be inadequate.

**Fig. 1:**
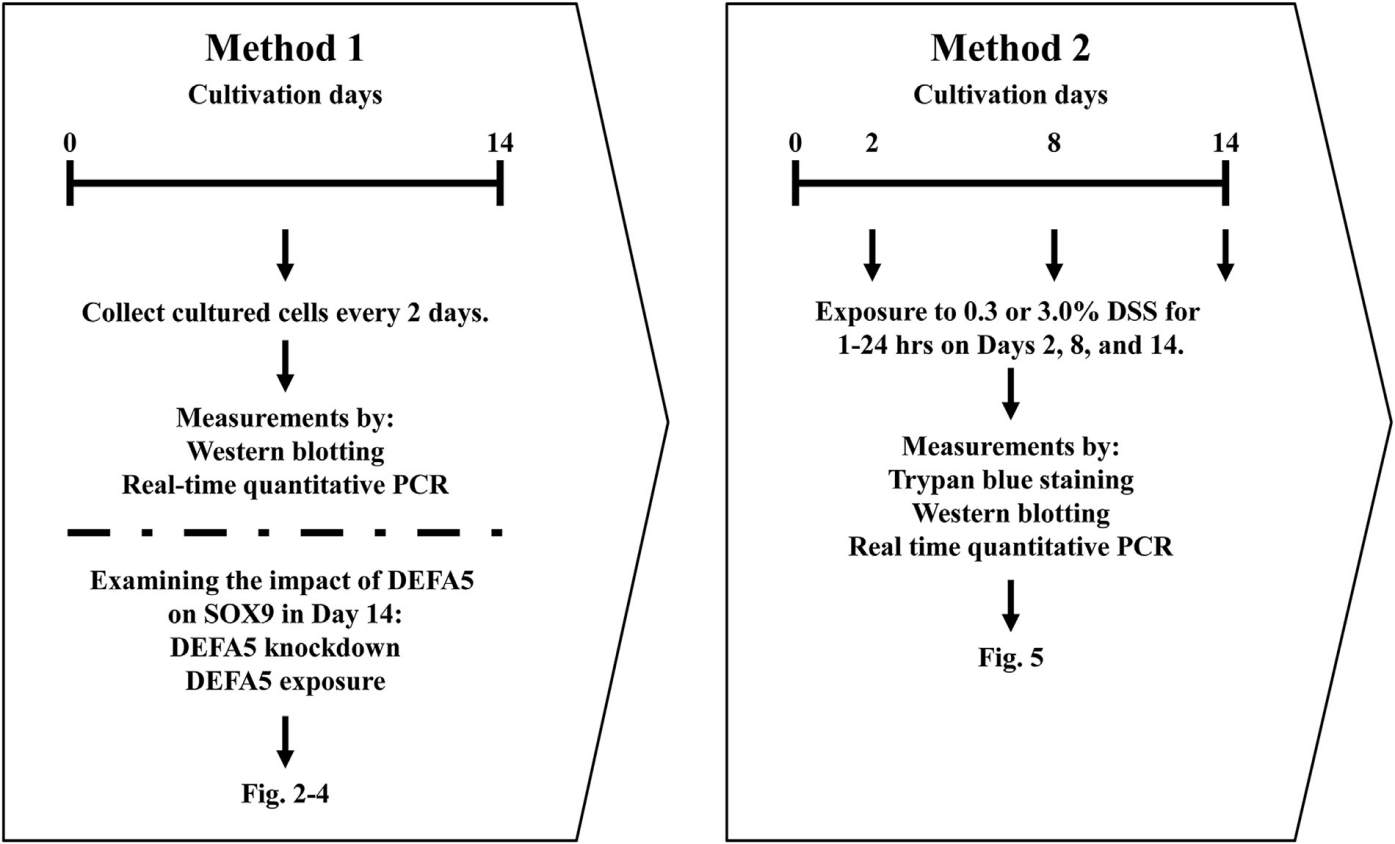
Overview of culture and cell collection methods. (Method 1) Cells were harvested on Days 2, 4, 6, 8, 10, 12, and 14 from a 14-day culture. Subsequently, mRNA and protein were extracted, and various indicators were measured. On Day 14, the cells were subjected to *DEFA5* knockdown or 18 μg/mL DEFA5 exposure for 1 h, followed by an assessment of fluctuations in SOX9 expression. (Method 2) On Days 2, 8, and 14 of a 14-day culture, the cells were subjected to 0.3 % or 3 % DSS exposure for 1 to 24 h. Subsequently, mRNA and protein were extracted, and survival indices were measured. Abbreviations: DEFA5, α-defensin5; SOX, SRY-box transcription factor; DSS, sodium dextran sulfate.

After 2, 4, 6, 8, 10, 12, and 14 days of culture, Caco-2 cells were collected. To ensure measurable cell volume, 6-well plates were used on days 2 and 4 of culture and 12-well plates on Day 6 and beyond. The buffer volume used for cell lysis was adjusted to ensure a uniform loading volume (1 µg/µL) in each well, especially considering western blotting, in parallel with protein quantification by the bicinchoninic acid assay (BCA) assay. After 2, 8, and 14 days of culture, Caco-2 cells were exposed to 0.3 % or 3 % DSS solutions dissolved in media for 1, 3, 6, and 24 h. Cell viability was assessed using trypan blue (Bio-Rad Laboratories, Hercules, CA, USA) staining by mixing in equal proportions with the suspension before protein or RNA extraction. For comparison with the knockdown experiment described below, cells were cultured for 14 days, then exposed to 18 μg/mL DEFA5 for 1 h to confirm the extracellular effects of the secreted protein DEFA5. A detailed schematic of the methods used in this study is presented in Fig. 1.

## Western blotting

Protein expression levels were determined as previously reported [10]. The control and treated cell lysates (10 µg protein/lane) were separated using 4–20 % sodium dodecyl sulfate-polyacrylamide gel electrophoresis (Bio-Rad Laboratories). Subsequently, the proteins were transferred onto a polyvinylidene difluoride membrane (Bio-Rad Laboratories) and subjected to western blotting using appropriate primary antibodies (Santa Cruz Biotechnology, Dallas, TX, USA) (Supplementary Table 1). The protein load was normalized through Stain-Free imaging of total protein [27]. Consistency was ensured between β-Actin as a loading control and total protein staining (Supplementary Fig. 1).

## Reverse transcription-quantitative polymerase chain reaction (RT-qPCR)

Gene expression levels were determined as previously reported [10]. In brief, total RNA was extracted from Caco-2 cells using ISOGEN reagent (NIPPON Genetics Co., Ltd., Tokyo, Japan). Single-stranded cDNA was synthesized from 1 µg of total RNA using ReverTra Ace (Toyobo, Osaka, Japan). Reverse transcription-quantitative polymerase chain reaction (RT-qPCR) was performed using the QuantStudio™5 real-time PCR system (Applied Biosystems, Foster City, CA, USA) with KAPA SYBR™ FAST (NIPPON Genetics Co, Ltd), according to the manufacturer's protocol. The final concentration of cDNA was 5 ng/µL, and of primers was 200 nM. The PCR cycling conditions were as follows: 40 cycles at 95 °C for 10 s, 50−60 °C for 34 s, and 72 °C for 1 s. The sequences of the specific primers used in this study are listed in Supplementary Table 2. The expression levels of the PCR products were normalized to that of the internal reference *ACTB*.

To prevent PCR inhibition by DSS, spermine (Sigma Aldrich, St. Louis, MO, USA) was used at a concentration < 0.01 g/L [28]. Given the use of reverse transcriptase in RT-PCR and real-time PCR, we examined the presence and absence of spermine at each stage. To confirm the restoration of enzyme activity by spermine, the Ct values of the housekeeping gene *ACTB* were compared with those of the target gene (Supplementary Table 3). Spermine is water-soluble, thus it was pre-dissolved in ultrapure water in each PCR mixture to facilitate homogenization.

## Transient transfection of small interfering RNA

DEFA5 siRNA (Oligo ID: HSS141812) and the negative control (NC) (Stealth™ RNAi negative control medium GC duplex) were purchased from Thermo Fisher Scientific. siRNA transfection was optimized using Lipofectamine™ RNAiMAX (Invitrogen, Carlsbad, CA, USA), according to the manufacturer's instructions. DEFA5 siRNA or NC (final concentration of 10 nM) and OPTI-MEM ™ reduced serum medium (Gibco, Grand Island, NY, USA) were mixed, followed by the addition of Lipofectamine™ RNAiMAX. Caco-2 cells (1.5 × 10^5^ cells/mL) were then suspended in a growth medium without antibiotics. After siRNA transfection (24 h), the cells were incubated as described in “Cell Culture and Treatment” for an additional 72 h before analysis. In detail, after 13 min of incubation at   20–22 °C a mixture of Opti-MEM (control), NC, or siRNA, and RNAi MAX was applied to plates in the following order: Control, NC, and siRNA. The cell suspension was applied in the order of NC, siRNA, and control by pipetting gently only once. The medium was mixed lightly and incubated for 24 h before replacing the medium. Notably, an aspirator was not used to avoid disturbing cell adhesion; a micropipette was used in all procedures. Protein and RNA samples collected in this process were analyzed for knockdown efficiency and expression levels using western blotting and RT-qPCR, respectively. The effects of the knockdown experiment on cell proliferation are summarized in Supplementary Fig. 2.

## Immunofluorescence microscopy of Caco-2 cell monolayers

Caco-2 cells were seeded at a density of 1.0 × 10^4^ cells/mL in μ-Slide 8 Wells (IBIDI GmbH, Martinsried, Germany). The culture medium was aspirated, and cells were washed three times using 0.1 % Tween20-PBS before fixation in 4 % paraformaldehyde (Fujifilm Wako Pure Chemicals, Osaka, Japan) at 20–22 °C for 10 min. Cells were then blocked with 0.1 % Triton-X (Sigma Aldrich, St. Louis, MO) and EzBlock Chemi (ATTO) for 60 min at 20–22 °C. Subsequently, the samples were incubated for 18 hrs at 4 °C with mouse monoclonal anti-SOX9 antibody (Santa Cruz Biotechnology, Dallas, TX, USA, catalog number: sc-166,505) diluted 100-fold in Can Get Signal Solution B (Toyobo, Osaka, Japan). After washing three times with 0.1 % Tween20-PBS for 5 min, Alexa Fluor 647-conjugated anti-mouse IgG secondary antibody (Santa Cruz Biotechnology, catalog number: sc-516,178) was diluted 500-fold and incubated at 20–22 °C for 1 hr. The same antibody dilution and incubation conditions were applied. Cells were washed an additional three times with 0.1 % Tween20-PBS for 5 min and reacted with Alexa Fluor 488-conjugated mouse monoclonal anti-Lysozyme antibody (Santa Cruz Biotechnology, catalog no sc-518,012) and Alexa Fluor 546-conjugated mouse monoclonal anti-α-defensin5 (Santa Cruz Biotechnology, catalog no sc-53,997) at a 100-fold dilution for 18 hrs at 4 °C. Finally, cells were washed three times with 0.1 % Tween20-PBS for 5 min and incubated with a 100-fold dilution of 4ʹ,6-diamidino-2-phenylindole (DAPI) for 1 hr at 20–22 °C. After removing DAPI, cells were examined with a laser scanning confocal microscope (LSM 700; Zeiss, Oberkochen, Germany) for imaging and evaluation. Results are shown in Supplementary Figure 3.

## Statistical analysis

All statistical analyses were performed using the Excel statistical software package (Bell Curve for Excel, Microsoft, Redmond, WA, USA). Statistically significant differences in means between two groups were evaluated using Student's *t*-test, and statistically significant differences in means between two more groups were evaluated using Dunnett's test. Statistical significance was set at *p* < 0.05 or 0.01.

## Method validation

### Setting differentiation levels in terms of Paneth cell-associated marker protein and mRNA expression levels

**Fig. 2:**
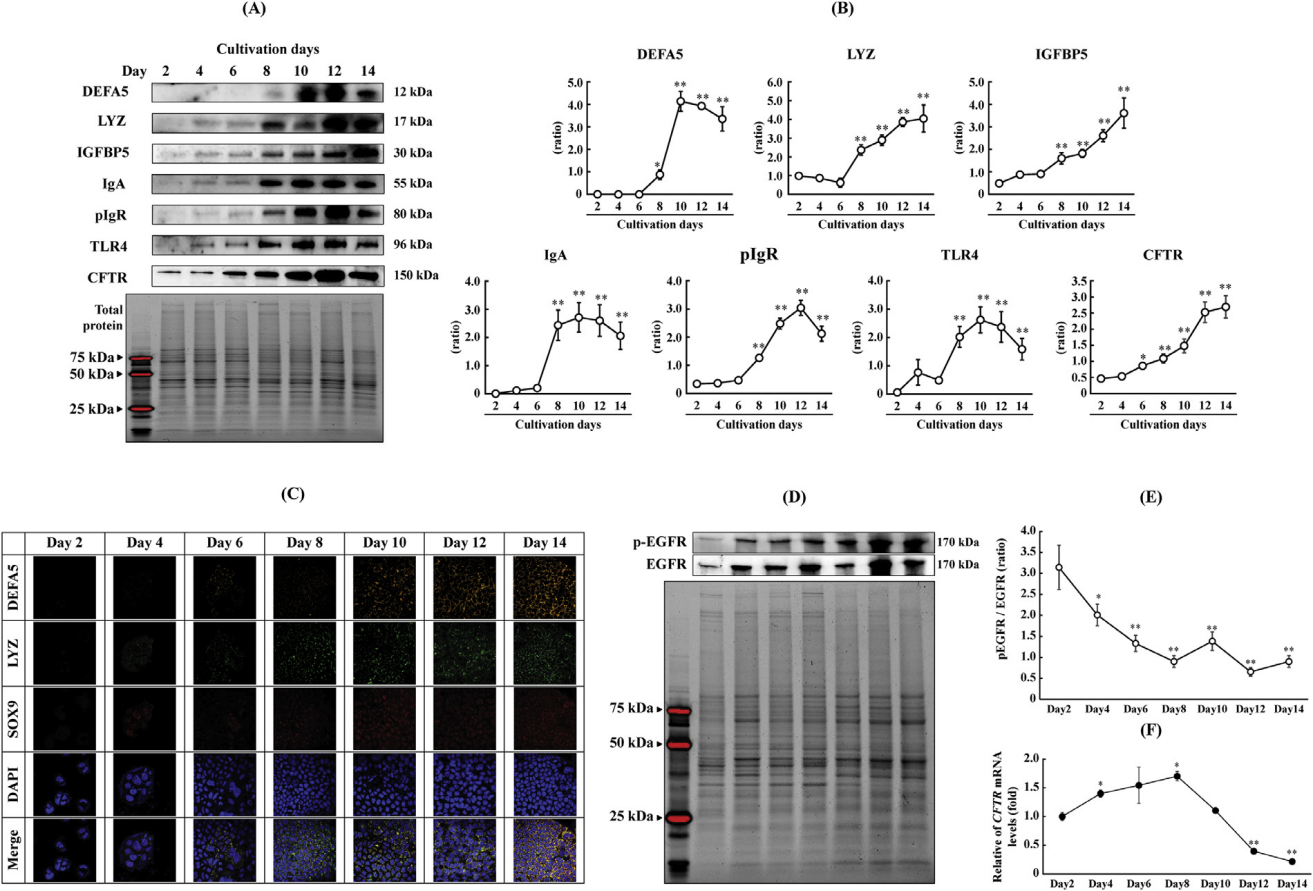
Changes in immune-related marker abundance throughout a 14-day incubation. (A,B) Western blotting analysis of protein levels of DEFA5, LYZ, IGF-BP5, IgA, pIgR, TLR4, and CFTR on Days 2, 4, 6, 8, 10, 12, and 14. (C) Immunostaining pf SOX9, LYZ and DEFA5 is shown. Localization of SOX9, DEFA5 and LYZ in Caco-2. Multiple immunofluorescent staining was performed for SOX9 (red), DEFA5 (orange), LYZ (green) and DAPI (blue). Only merged images of nuclear staining and each protein staining are shown. Scale bars: 40 μm. (D,E) Western blot analysis of the phosphorylation level of EGFR on Days 2, 4, 6, 8, 10, 12, and 14. (F) qRT-PCR analysis of *CFTR* mRNA expression on Days 2, 4, 6, 8, 10, 12, and 14. (B) Data were analyzed using Dunnett's test. *p < 0.05 and **p < 0.01 compared with the Day 2 group. Data are presented as means ± SD from three independent experiments (*n* = 3). The protein load was normalized using Stain-Free imaging of total protein. Abbreviations: DEFA5, α-defensin5; LYZ, lysozyme; IGF-BP5, insulin-like growth factor binding protein 5; IgA, immunoglobulin A; pIgR, polymeric immunoglobulin receptor; TLR4, toll-like receptor 4; EGFR, epidermal growth factor receptor; CFTR, cystic fibrosis transmembrane conductance regulator; SD, standard deviation; SOX9, SRY-box transcription factor 9; LYZ, Lysozyme; DAPI, 4′,6-diamino-2-phenylindole.

In their evaluation of Paneth cells using Caco-2 cells, Yasuda *et al*. reported that DEFA5 secretion increases along a positive slope for up to 14 days, exhibiting plateau fluctuation from Day 14 on [6]. Accordingly, we followed the fluctuations of various markers, including DEFA5, on Caco-2 cells under normal culture conditions for up to 14 days to determine the day from which expression was observed. Stimulating LGR5-positive stem cells with WNT3 induces their differentiation into mature Paneth cells expressing lysozyme and DEFA5 via SOX9-dependent signaling [[17], [18], [19]]. Given that lysozyme is expressed in immature Paneth cells, whereas DEFA5 is expressed only in mature Paneth cells [20], DEFA5 was used as an indicator of cell differentiation [29]. Proteins were collected every 2 days from Days 2 to 14, and their expression levels were assessed. The expression of DEFA5, IgA, lysozyme, and IGF-BP5 was detectable from Day 8 of culture (Fig. 2A,B). As shown in Fig. 2C and Supplemental Fig. 3, immunostaining results also confirmed a cell morphology in which LYZ expression was predominant at Day 8, and DEFA5 expression was also observed from Day 10 to 14. Similarly, the expression of TLR4—involved in inducing DEFA5 and other proteins—and pIgR—involved in IgA secretion—was detectable from Day 8 onward (Fig. 2A). Meanwhile, the abundance of phosphorylated epidermal growth factor receptor (EGFR) exhibited a decreasing trend after Day 8, suggesting a predominance of differentiated cells (Fig. 2D,E).

As reported in our previous study, DEFA5 induces the expression of chloride channel cystic fibrosis transmembrane conductance regulator (CFTR), which is involved in maintaining the activity of antimicrobial peptides, by maintaining salinity and water content on the luminal side [10]. Moreover, variations were observed in mRNA expression throughout the incubation period. For example, a gradual decrease in *CFTR* mRNA expression was initially observed, peaking on Day 8 (Fig. 2F). Conversely, protein expression increased on Day 8 (Fig. 2B). This highlights the need to consider differentiation stage-dependent changes in the post-secretory activity of DEFA5. The tumor necrosis factor-α (TNF)/CFTR signaling axis reportedly contributes to mucin homeostasis in the intestinal tract [30]. Meanwhile, we previously reported on the CFTR regulatory mechanism by DEFA5 [10], providing insights that may facilitate a differentiation stage-based assessment to elucidate the communication between mucin-secreting goblet cells and DEFA5-secreting Paneth cells.

According to our results, Day 8 denotes early differentiation, characterized by lysozyme and DEFA5 expression. Meanwhile, the differentiation and maturation of Paneth-like cells in Caco-2 cells occurs between Days 8 and 14. Notably, the chloride channel CFTR, which is targeted by DEFA5 [10], exhibited synchronized expression levels during this period, independent of mRNA expression. Furthermore, compared to the findings of a previous study, which assessed the activity and secretory capacity of alkaline phosphatase and DEFA5, in the present study, alkaline phosphatase and DEFA5 expression tended to increase from Day 14, ultimately plateauing and supporting Paneth-like cell differentiation [6].

**Fig. 3:**
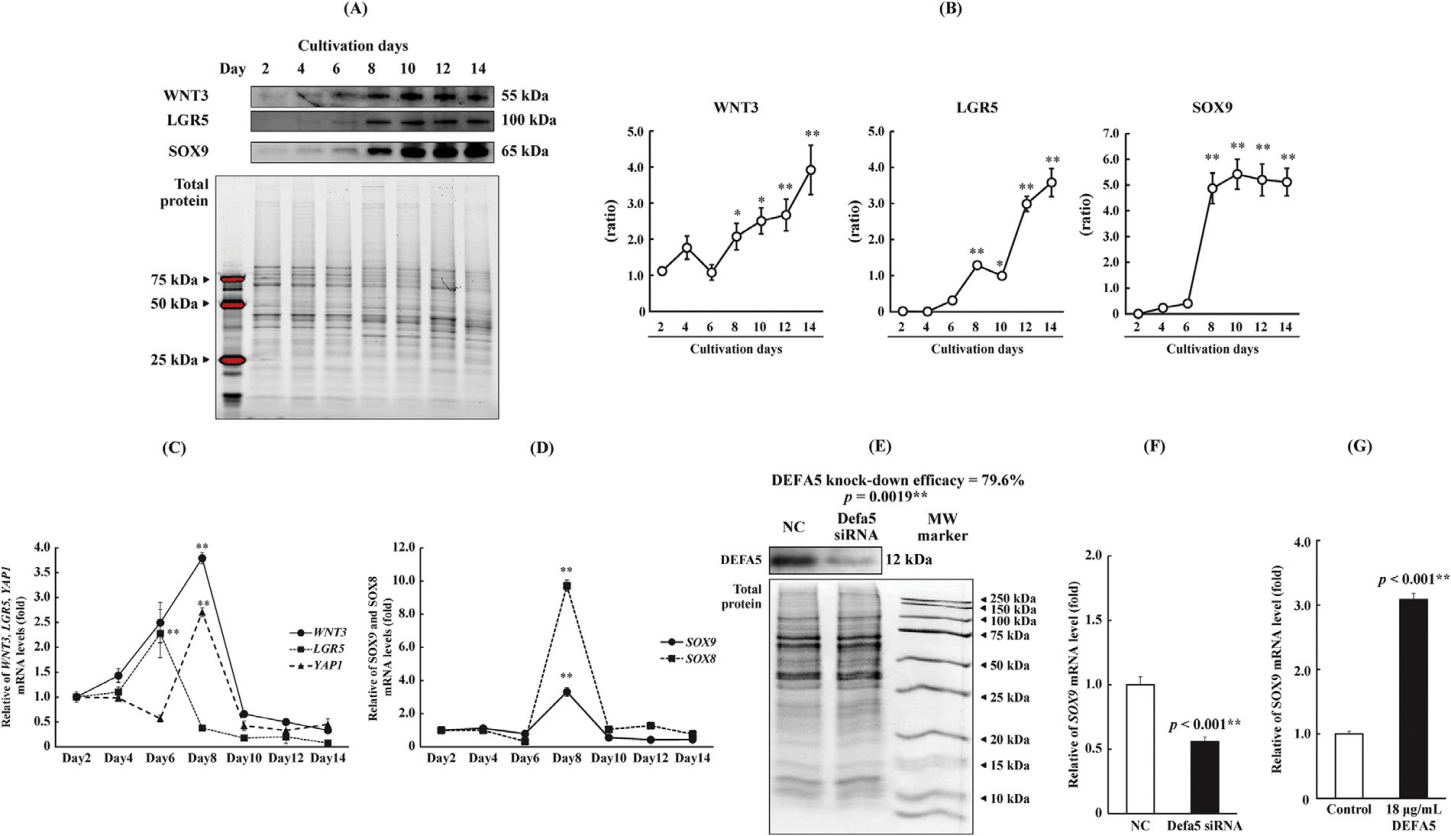
Changes in protein and gene expression of differentiation-related markers throughout a 14-day culture. (A.B) Western blot analysis of WNT3, LGR5, and SOX9 protein levels on Days 2, 4, 6, 8, 10, 12, and 14. (C.D) qRT-PCR analysis of *WNT3, LGR5, YAP1, SOX9*, and *SOX8* mRNA expression on Days 2, 4, 6, 8, 10, 12, and 14. (E) DEFA5 knockdown efficacy. (F) *SOX9* gene expression in *DEFA5*-deficient cells. (G) *SOX9* mRNA expression after 1-h exposure to DEFA5. Data are presented as means ± SD from three independent experiments (*n* = 3). The protein load was normalized using Stain-Free imaging of total protein. (E-G) Data were analyzed using Student's *t*-test. *p < 0.05 and **p < 0.01 compared with the control group. (B) Data were analyzed using Dunnett's test. *p < 0.05 and **p < 0.01 compared with the Day 2 group. Abbreviations: *WNT3*, Wnt family member 3; *LGR5*, leucine-rich repeat-containing G protein-coupled receptor 5; *SOX*, SRY-box transcription factor; *YAP1*, yes1 associated transcriptional regulator 1; DEFA5, α-defensin5; SD, standard deviation.

We also isolated proteins and mRNA from cultured cells and assessed the expression of differentiation markers. The expression of LGR5, WNT3, and SOX9 was confirmed from Day 8 onwards, mirroring the pattern observed for antimicrobial peptides (Fig. 3A,B). The induction of mRNA expression of the investigated genes was completed by Day 8 (Fig. 3C). Additionally, *SOX9—*a marker of Paneth cell maturation—exhibited a significant increase in expression on Day 8 (Fig. 3D). Furthermore, the expression of *SOX8*—a recognized M-cell differentiation marker—was detected in Caco-2 cells, exhibiting a significant increase in expression simultaneously with *SOX9* (Fig. 3D).

Moreover, the association between IBD and the transcription factor SOX9 has received considerable research attention, with studies suggesting its involvement in disease regulation [31]. To assess the effect induced by DEFA5 on SOX9, the relationship between SOX9 and DEFA5 in Paneth cells was examined by knockdown experiments. Alpha-defensin5 knockdown decreased *SOX9* expression, whereas the addition of DEFA5 induced transcription, suggesting that Paneth cell-derived DEFA5 induces the differentiation of adjacent Paneth cells (Fig. 3E-G; *p* < 0.01). Moreover, *YAP1* expression increased significantly on Day 8, indicating that this time point is an important juncture for cell differentiation and proliferation (Fig. 3C). Moreover, a rapid increase in the proliferation of Caco-2 cells was observed from Day 8 (Supplementary Fig. 3). Considering the fluctuations in the protein expression of differentiation markers, we designated Day 8 as the early stage of differentiation in this study. These results suggest that when replicating the differentiation of Paneth cells in Caco-2 cells, the period preceding Day 8 can be considered the undifferentiated stage, Day 8 as an early differentiated stage, and Day 14 as a mature stage.

In addition to an increase in the levels of antimicrobial substances derived from Paneth cells, the expression of IgA and IGF-BP5 increased around Day 8. In the testis, SOX8 and SOX9 act in a coordinated manner during sexual differentiation [32,33]. Meanwhile, in the intestine, SOX8 and SOX9 function as differentiation markers for M and Paneth cells, respectively [17,34]. Therefore, further investigation into the synchronization of SOX8 and SOX9 expression observed in Caco-2 cells may advance research on the acquired immune system. Notably, it has been suggested that the substances necessary for differentiation are also secreted into the culture medium at this time point [35].

## Validation based on immune responses to DSS exposure according to differentiation level

**Fig. 4:**
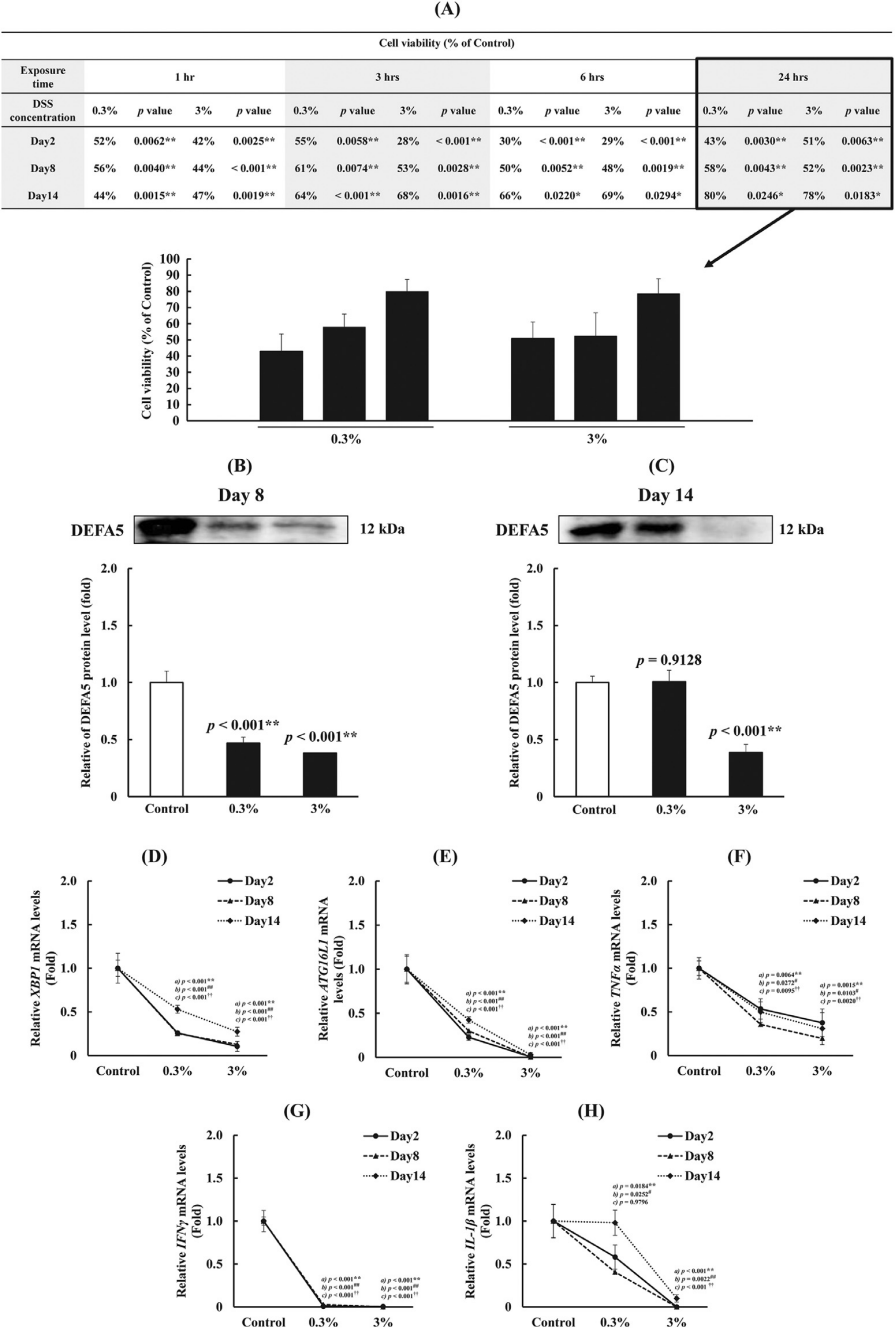
Immune response according to the level of cell differentiation induced by DSS exposure. (A) Variations in cell viability upon DSS exposure on Days 2, 8, and 14 (temporal and concentration observations). (B) Variations in DEFA5 protein expression in response to DSS exposure on Day 8. (C) Variations in DEFA5 protein expression in response to DSS exposure on Day 14. Data were analyzed using Dunnett's test. * *p* < 0.05 and **p < 0.01 compared with the control group. (D–H) Changes in *XBP1, ATG16L1, TNFA, IFNG*, and *IL1B* expression in response to DSS exposure on Days 2, 8, and 14. *a)* * *p* < 0.05 and **p < 0.01, Day 2 group compared to the control group. *b)* † *p* < 0.05 and ††*p* < 0.01, Day 8 group compared to the control group. *c)* #*p* < 0.05 and ##*p* < 0.01, Day 14 group compared to the control group. Data are presented as mean ± SD from three independent experiments (*n* = 3). Abbreviations: DEFA5, α-defensin5; DSS, dextran sulfate sodium; *XBP1*, X-box binding protein 1; *ATG16L1*, autophagy-related 16 like 1; *TNFA*, tumor necrosis factor-α; *IFNG*, interferon-γ; *IL1B*, interleukin 1β; SD, standard deviation.

Based on the designated culture days for each differentiation stage, the DSS results were validated by exposure experiments (Method 2 in Fig. 1). Initially, cell viability uniformly decreased in a time-dependent manner in response to DSS exposure on Days 2 and 8; however, on Day 14, cells were less susceptible to the DSS-induced decrease in viability (Fig. 4A). To further investigate the initial response at 1 h after DSS exposure, the changes in DEFA5 expression were measured. However, DEFA5 bands were not detectable on Day 2. Although its expression was suppressed by 0.3 % DSS on Day 8 (Fig. 4B), no suppression occurred when exposed to DSS at low concentrations on Day 14 (Fig. 4C). The alterations in cell viability induced by DSS generally align with previously reported findings, facilitating discussion while excluding osmotic-dependent cytotoxicity attributed to changes in DSS concentration [13]. Following IBD induction by DSS, disruption of tight junctions and an increase in ectopic Paneth cell number outside the intestinal crypts have been observed [36,37].

Furthermore, exposure to DSS at higher concentrations and longer durations may increase DSS persistence in cells, enhancing the inhibitory effect. This study confirmed the effect of DSS on the expression of autophagy-related 16-like 1 (*ATG16L1*) and X-box binding protein-1 (*XBP1*), genes used to evaluate the innate immune system, including DEFA5, in enteritis models [20,38]. There was a significant decrease in their expression following exposure to DSS in a dose-dependent manner on Days 2, 8, and 14 (Fig. 4D, E; *p* < 0.01). In contrast, this suppressive effect of DSS tended to decrease with increasing culture duration (Fig. 4D, E), indicating the importance of suppressing the expression of enteritis-related factors during immaturity and early differentiation. We also confirmed the effects of DSS on the expression of select cytokines *TNFA*, interleukin-1β (*IL1B*), and interferon-γ (*IFNG*), with significant inhibition of *TNFA* and *IFNG* expression (Fig. 4-F, G; *p* < 0.05) and relatively complete inhibition of *IFNG* expression. In contrast, *IL1B* exhibited a significant concentration-dependent decrease in expression on Day 2, followed by an increase on Day 14 at low concentrations, similar to the DEFA5 protein expression pattern (Fig. 4-H; *p* < 0.05). Since IL-1β, like DEFA5, is produced in Paneth cells, a differentiation-dependent immune response in Paneth cells was likely expressed. Taken together, these results validate that the DEFA5-dependent defense mechanism is a differentiation stage determinant of survival and immune responsiveness to DSS.

Our results on the effect of DEFA5 to induce SOX9 expression, variations in DEFA5 expression in response to DSS at different stages of differentiation, and fluctuations in survival rates suggest a direct correlation between these factors and the differentiation process in the early stages of IBD induction. This correlation may be closely linked to alterations in host defense mechanisms following the remission of the disease state. Moreover, DSS exposure suppressed the transcription of *XBP1* and *ATG16L1* [20,39], which participate in DEFA5 secretion. Additionally, given that DSS reportedly induces inflammation in intestinal tissues in vivo [[3], [4], [5]], we initially assumed that exposure to DSS in Caco-2 cells would increase inflammatory cytokine levels. However, our results indicated suppressed transcription of all tested cytokines, excluding *IL1B*, upon exposure to DSS at low concentrations. A notable difference between DSS-treated model mice and Caco-2 cells is the absence of immune cell populations, such as macrophages in Caco-2 cells. Overall, these results suggest the successful establishment of an *in-vitro* model of IBD.

In the intestinal immune system, crosstalk occurs among microflora, host tissues, and blood cell components [35]. This interaction has been demonstrated by in vivo models that have elucidated mechanisms such as hydrogen sulfide generation and inflammation triggered by the metabolism of intestinal microflora in the presence of DSS. Moreover, intestine-derived IL-33 and IL-7 impact intestinal lymphocyte and B cell activation. The results of the present study, together with those of previous studies, suggest that immunosuppression in Caco-2 cells targets specific elements in the intestinal endemic tissue. Specifically, aberrant signaling to macrophages and other cells by Paneth cells, goblet cells, and other secretory substances derived from intestinal cells may ultimately induce excessive cytokine production. While *in-vitro* models such as Caco-2/HT-29 co-cultures can simulate in vivo conditions and aid in testing hypotheses [23], the differentiation stages of intestinal cells pose a challenge owing to proliferation rate differences. Thus, validating our results using Caco-2 cells, which exhibit slower differentiation than HT-29 cells, is vital for accurately modeling IBD pathology.

## Limitations

Differences in human regulatory mechanisms in animal models, even relatively human-like animals, may be due to differences in the subtypes of protein and the proportion of expressed molecules [20,21]. Although research has been conducted on various types of IBD, the therapeutic targets have been limited to suppressing inflammation [22] with the aim of achieving symptom remission. No curative targets have been discovered [3], necessitating methods to collect human cell-derived information to help discover new therapeutic targets. We sought a way to assess cell differentiation defects in IBD pathology with a model that could reliably observe the differentiation process of Paneth cells. In addition, as we are in the process of establishing an experimental system to observe the effects of DSS exposure in the undifferentiated state on post maturity of cells, our study, showing variations in gene and protein expression with culture duration and differentiation stage, is crucial; however, this study was limited by the lack of assessment of signaling from intestinal tissues to blood cell components. The expression of inflammatory cytokines such as IL-10 may also be suppressed, which needs to be verified by co-culturing Caco-2 cells with hematopoietic cells, as reported by Tanoue et al. [40] For this purpose, the differentiation stages defined by us can be used to further analyze cell–cell interactions according to each stage of differentiation. In future studies, we will conduct knockdown experiments to confirm the role of DEFA5 and other gut-derived substances in crosstalk and to elucidate the potential actions of DEFA5 and other antimicrobial peptides. Given the considerable time, resources, and ethical issues involved in developing animal models of IBD using DSS, the establishment of this in-vitro system using Caco-2 cells is promising for the advancement of IBD research. However, unlike proliferative cells such as Caco-2 cells, primary human cell lines have a limited number of passages and are not suitable for screening; moreover, these cells may lack versatility due to variations in target protein expression caused by variations in the stability of experimental methods and the limitations of each research facility in securing human samples.

3D organoid cultures and 2D organoid monolayer cultures represent the gut crypts more accurately because of their mimicry of complex tissue structures and cell–cell interactions; however, certain practical considerations justified the use of cell lines in the present study. Such sophisticated models can be challenging to use because of variability in initial culture conditions and reagents among laboratories, leading to inconsistencies and impeding the standardization of protocols. Additionally, establishing and maintaining 3D organoid cultures and 2D organoid monolayers can be resource-intensive, whereas cell lines are more practical and cost-effective for screening purposes. Differentiation in cell lines can be simply defined in terms of days under normal culture conditions, allowing for more consistent and reproducible results suitable for high-throughput screening and early exploratory studies. The use of cell lines also facilitates the rapid and efficient screening of reagents and conditions, providing a fundamental understanding that can be subsequently validated and developed using more complex organoid models. However, it is crucial to acknowledge the limitations of using Caco-2 cells. While these cells are commonly used because of their ability to differentiate and mimic the intestinal epithelial barrier, they do not fully replicate the complexity and cellular diversity of the human intestinal epithelium. This lack of diversity can result in differences in cell signaling, metabolism, and transport mechanisms, compared to primary cells or tissues. Additionally, Caco-2 cells frequently exhibit altered differentiation patterns and may not express all enzymes and transporters that occur in normal enterocytes. Their cancerous origin also implies that they may behave differently from non-cancerous cells in terms of proliferation, gene expression, and response to stimuli. These factors limit the extent to which findings using Caco-2 cells can be generalized to in vivo conditions. Despite these limitations, the use of Caco-2 cells allowed us to perform initial screening efficiently and cost-effectively, providing a foundation for subsequent validation using more complex models such as 3D organoids and 2D monolayer cultures.

Furthermore, animal models for pathological conditions can be constructed through genetic modifications or via external induction of inflammation through long-term administration of reagents such as DSS; however, in the former approach, pathogenesis has already been determined while confirming the junction point between intestinal cell differentiation and pathological development is challenging. Whereas, in the latter approach, it is difficult to confirm each stage of cell differentiation in response to reagents. It is also limited by the unknown sensitivity of the cell population at each differentiation stage to reagents. Therefore, this method provides an important basis for fundamental studies at the midpoint between single-cell analysis and organ-level analysis. Moreover, this system will be useful for not only elucidating pathogenesis, but also identifying therapeutic targets. For example, further investigation of the effects of Caco-2 cells on the defense mechanisms of intestinal stem cells during early differentiation may lead to the discovery of novel therapeutic targets specific to humans. In addition, epigenetic inheritance has been reported in IBD [41]; thus, analysis of the promoter regions of human target genes is needed in mouse models to identify genes that are not affected by DNA methylome analysis [42].

The present study supplements a previous study that sought to identify food ingredients and drugs capable of counteracting causative factors of IBD and reducing the incidence of IBD through continuous screening of patients for the causative factors. This proactive approach is consistent with our goal of mitigating the effects of IBD and improving patient outcomes.

## Ethics statements

Not applicable.

## CRediT authorship contribution statement

**Ippei Uemura:** Conceptualization, Formal analysis, Investigation, Writing – original draft, Writing – review & editing, Visualization. **Natsuko Takahashi-Suzuki:** Conceptualization, Investigation, Supervision, Writing – original draft, Writing – review & editing, Funding acquisition. **Fumiya Kita:** Investigation. **Takashi Satoh:** Supervision, Writing – review & editing.

## Declaration of competing interest

The authors declare that they have no known competing financial interests or personal relationships that could have appeared to influence the work reported in this paper.

## Data Availability

Data will be made available on request.

## Appendix Supplementary materials

Image, application 1
