# Supplementary material for: Establishment of an in-vitro inflammatory bowel disease model using immunological differentiation of Caco-2 cells
Source: MethodsX. 2024 Sep 13;13:102952. doi: 10.1016/j.mex.2024.102952 (PMC11426153; doi:10.1016/j.mex.2024.102952)
Supplement: Supplementary file 1 [file mmc1.docx]

Uemura et al.

Supplementary Materials

**Supplementary Table 1.** Antibodies used in this study

| Antibody | Manufacturer | Catalog # | Lot No. | Dilution |
| --- | --- | --- | --- | --- |
| α-defensin5 (8c8) | Santa Cruz Biotechnology, Dallas, TX, USA | SC-53997 | #A0917 | 1:250 |
| Lysozyme C (E-5) |  | SC-518012 | #G1620 | 1:500 |
| IGFBP5 (D-6) |  | SC-515116 | #J2021 | 1:500 |
| IgA (A-9) |  | SC-373823 | #A2820 | 1:500 |
| SC (C-2) |  | SC-374343 | #H0117 | 1:500 |
| TLR4 (25) |  | SC-293072 | #C0123 | 1:500 |
| CFTR (A-3) |  | SC-376683 | #F0220 | 1:500 |
| Wnt-3 (D-9) |  | SC-74537 | #L1119 | 1:500 |
| LGR5 (634J2E) |  | SC-517661 | #J1520 | 1:500 |
| Sox-9 (E-9) |  | SC-166505 | #F0123 | 1:500 |
| EGFR (A-10) |  | SC-373746 | #I1714 | 1:500 |
| m-IgGκ BP-HRP |  | SC-516102 | #A1023 | 1:1000 |
| m-IgG_2b_ BP-HRP |  | SC-542741 | #F2221 | 1:1000 |
| Mouse anti rabbit IgG-HRP |  | SC-2357 | #L1322 | 1:1000 |
| α-defensin5 (8c8) Alexa Fluor 546 |  | SC-53997 | #J0521 | 1:100 |
| Lysozyme (E-5) Alexa Fluor 488 |  | SC-518012 | #I1421 | 1:100 |
| Goat pAb to Ms IgA  (Alexa Fluor 647) | Abcam Inc, Toronto, ON, Canada, C | ab150115 | GR309891-1 | 1:500 |
| Phospho-EGF Receptor (Tyr1068) (D7A5) XP® Rabbit mAb | Cell Signaling Technology, Cambridge, UK | #3777 | 10 | 1:500 |

Abbreviations: IGFBP5, Insulin-like growth factor binding protein 5; IgA, Immunoglobulin A; SC, secretory component: TLR4, toll-like receptor 4; CFTR, cystic fibrosis transmembrane conductance regulator; Wnt-3, Wnt family member 3; LGR5, Leucine rich repeat containing G protein-coupled receptor 5; Sox-9, SRY-box transcription factor 9; EGFR, epidermal growth factor receptor

**Supplementary Table 2.** Primers used in this study

| Gene |  | Sequence (5′to 3′) | T_m_  (°C) | Product length (bp) | Annealing temperature (°C) |
| --- | --- | --- | --- | --- | --- |
| ACTB  NM_001101.4 | Fw | TGGCACCAGCACAATGAA | 57.40 | 185 | 60 |
|  | Rev | CTAAGTCATAGTCCGCCTAGAAGCA | 61.70 |  |  |
| WNT3  NM_030753.5 | Fw | CACCATTTGCGGCTGTGACT | 61.52 | 172 | 60 |
|  | Rev | CGCCTCGTTGTTGTGCTTGT | 62.05 |  |  |
| LGR5  NM_003667.3 | Fw | CAGGGTCTTCACCTCCTACC | 52.00 | 128 | 50 |
|  | Rev | TGGGAATGTATGTCAGAGCG | 48.00 |  |  |
| SOX9  NM_000346.4 | Fw | ATCTCCCCCAACGCCATCTT | 61.28 | 173 | 55 |
|  | Rev | TCTCGCTTCAGGTCAGCCTT | 61.19 |  |  |
| SOX8  NM_014587.5 | Fw | GCTCCCTCTCCCTTCTATCTTTCT | 60.94 | 136 | 63 |
|  | Rev | TGCCGTTTCTCCTCTACCGA | 60.61 |  |  |
| YAP1  NM_001130145.3 | Fw | GTTACCAACACTGGAGCAGGA | 59.93 | 53 | 55 |
|  | Rev | CGGGAGAAGACACTGGATTTTG | 59.26 |  |  |
| CFTR  NM_000492.4 | Fw | ACGTTGAAAGCAGGTGGGAT | 59.89 | 181 | 60 |
|  | Rev | AAGCCACTATCACTGGCACT | 59.01 |  |  |
| XBP1  NM_001079539.1 | Fw | AACCAGGAGTTAAGACAGCGCTT | 62.40 | 118 | 55 |
|  | Rev | CTGCACCCTCTGCGGACT | 61.70 |  |  |
| ATG16L1  XM_005246082 | Fw | ACGTACCAAACAGGCACGAG | 60.60 | 239 | 55 |
|  | Rev | CAGGTCAGAGATAGTCTGCAAAC | 58.80 |  |  |
| TNFα  NM_000594.4 | Fw | TCTTCTCGAACCCCGAGTGA | 60.30 | 164 | 57 |
|  | Rev | ATGAGGTACAGGCCCTCTGA | 59.70 |  |  |
| IFNγ  NM_000619.3 | Fw | AGATCCCATGGGTTGTGTGT | 58.92 | 124 | 50 |
|  | Rev | GCCATTAAAGCACTGGCTCA | 58.82 |  |  |
| IL-1β  NM_000576.2 | Fw | AGGAGAATGACCTGAGCACC | 50.00 | 320 | 60 |
|  | Rev | ATCATCTTTCAACACGCAGG | 45.90 |  |  |

Abbreviations: ACTB, β-actin; WNT3, Wnt family member 3; LGR5, Leucine rich repeat containing G protein-coupled receptor 5; SOX, SRY-box transcription factor; YAP1, Yes1 associated transcriptional regulator 1; CFTR, cystic fibrosis transmembrane conductance regulator; XBP1, X-box binding protein 1; ATG16L1, autophagy-related 16 like 1; TNFα, tumor necrosis factor-α.; IFNγ, interferon-γ; IL-1β, interleukin 1β; OSR, Odd-skipped related transcription factor; RIP, Receptor-interacting protein; Fw, forward; Rev, reverse; T_m_, melting temparature

**Supplementary Table 3.** Verification of reverse transcriptase activity recovery by spermine

| **DSS** | | + | + | － | + | **Student's**  ***t*-test** | **Welch's**  ***t*-test** |
| --- | --- | --- | --- | --- | --- | --- | --- |
| **RT-PCR** | **Spermine** | － | + | + | + |  |  |
| **Real time-PCR** | **Spermine** | － | － | － | + |  |  |
| **Ct value of *ACTB*** | **No.1** | Undetermined | Undetermined | 19.53915977 | 19.32495308 | 0.098997376 | 0.10216941 |
|  | **No.2** | Undetermined | Undetermined | 19.51841927 | 19.33618355 |  |  |
|  | **No.3** | Undetermined | Undetermined | 19.39898109 | 19.43546677 |  |  |

Data are presented as mean ± SD from three independent experiments (n = 3). Abbreviations: ACTB, β-actin; DSS, dextran sulfate sodium.

| (A) | (B) |
| --- | --- |
| 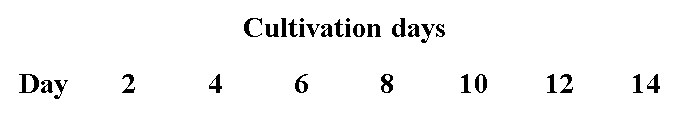  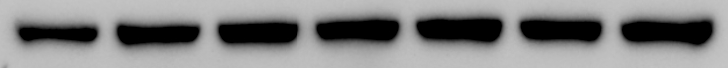  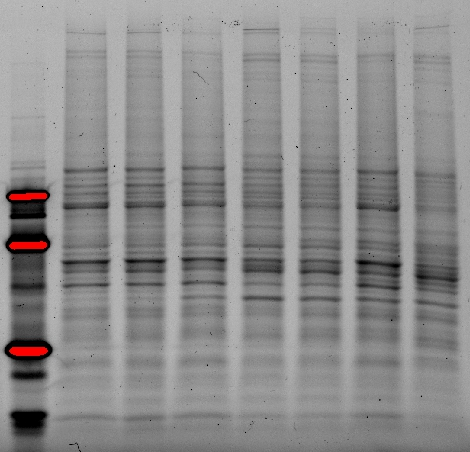 | 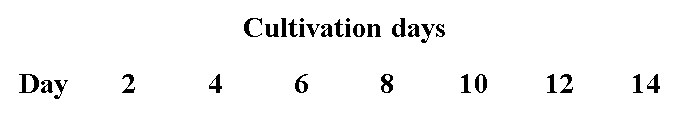  **ACTB**  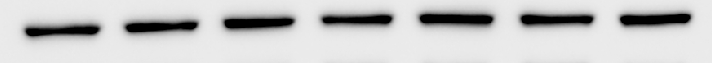  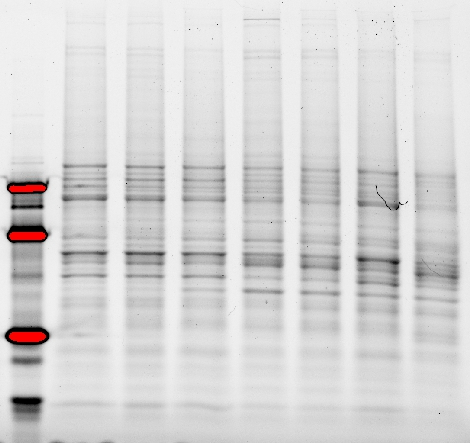 |
| (C) | (D) |
| 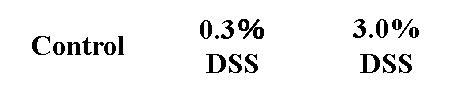  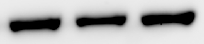  **ACTB**  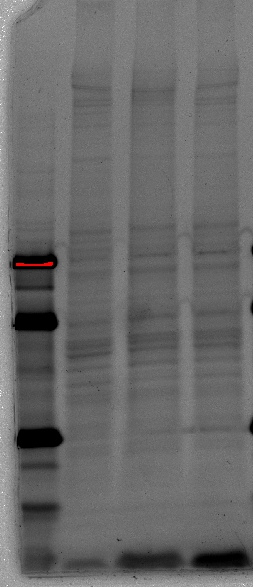 | 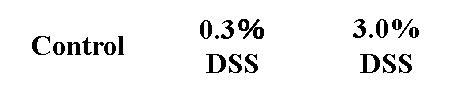  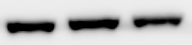  **ACTB**  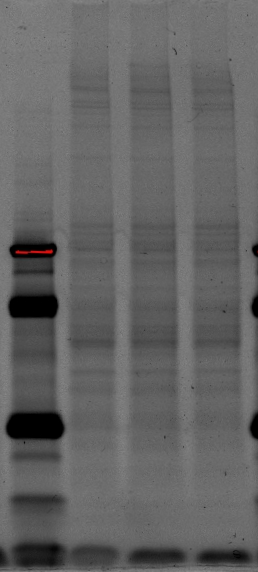 |

**Supplementary Fig. 1.** Reliable housekeeping proteins used in western blotting.

**ACTB**

ACTB band and image of stain-free gel for data presented in (A) Fig. 2A, (B) Fig. 3A, (C) Fig. 4B, and (E) Fig. 4C.

| **(A)** | | |
| --- | --- | --- |
| **Control** | **Negative Control** | **siRNA** |
|  |  |  |
| **(B)** | | |
| **NS** | | |

**Supplementary Fig. 2.** Confirmation of effect on cell proliferation by knockdown experiments.

(A) Caco-2 cells during the knockdown experiment, comparing the proliferative state of the Caco-2 cells. (magnification, 40×, Scale bar = 200 μm). (B) Changes in survival during knockdown experiments (trypan blue staining). Data were analyzed using Dunnett’s test. Data are presented as means ± SD from three independent experiments (n = 4). Abbreviations: NS, Not Significant; SD, standard deviation.

| **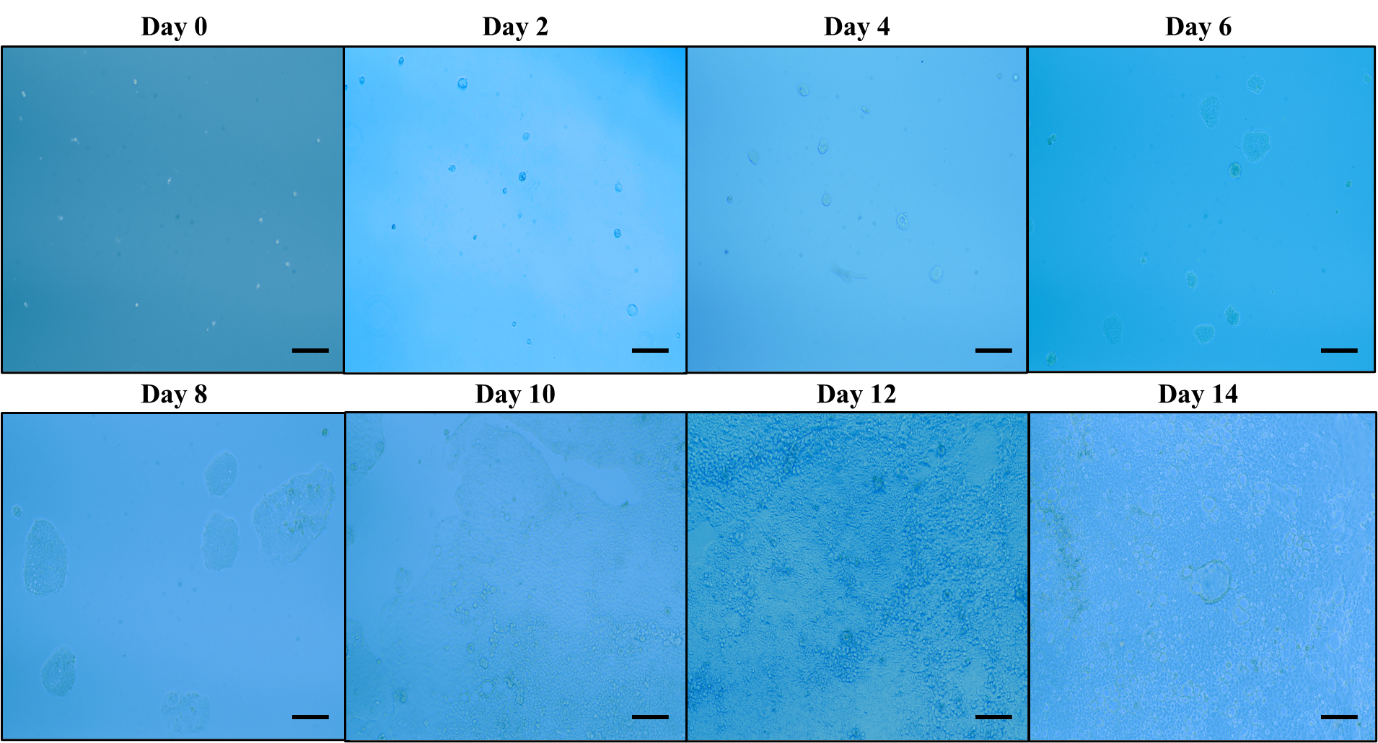** |
| --- |

**Supplementary Fig. 3.**  Progress of Caco-2 culture confirmed by optical microscopy.

Cell growth observed when the medium was changed on Days 2, 4, 6, 8, 10, 12, and 14 (magnification, 40×, Scale bar = 200 μm).
